# Supplementary material for: Cul3 regulates cytoskeleton protein homeostasis and cell migration during a critical window of brain development
Source: Nat Commun. 2021 May 24;12:3058. doi: 10.1038/s41467-021-23123-x (PMC8144225; doi:10.1038/s41467-021-23123-x)
Supplement: Supplementary file 1 — Supplementary information [file 41467_2021_23123_MOESM1_ESM.pdf]

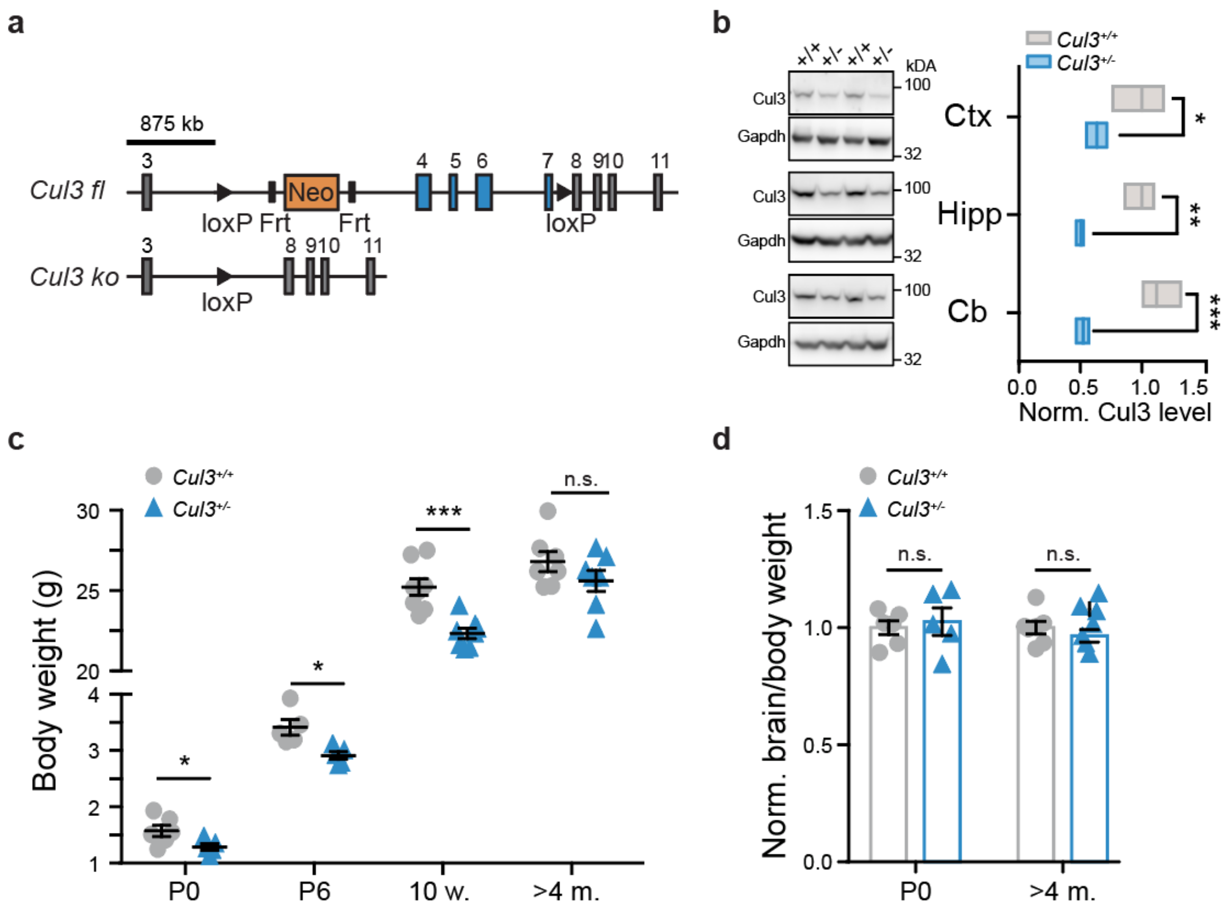

Supplementary Figure 1 The conditional *Cul3* allele and its deletion in mice.
a, Scheme of the conditional *Cul3* allele in mice in which exons 4-7 are flanked by two loxP sites (*Cul3*^{fl}). Cre-mediated recombination leads to genomic excision of the flanked region resulting in the *Cul3* knockout (*Cul3*^{ko}) allele. **b**, Representative Western blots and analysis of adult cortex (Ctx), hippocampus (Hipp) and cerebellum (Cb) reveal significantly decreased Cul3 levels in all brain regions of *Cul3*$^{+/-}$ mice (*n*= 3 per genotype; *P=0.049, **P=0.002, ***P=0.004; unpaired two-tailed t-tests). **c**, *Cul3*$^{+/-}$ mice are born with reduced body weight as compared to their wild-type littermates, a growth defect persisting until early adulthood (P6 and 10 weeks), but recovered at 4 month of age (*n*(P0, P6, 10w., 4m.)= 6, 5, 8, 7 per genotype respectively; *P=0.044 (P0), *P=0.012 (P6), ***P<0.001 (10w.), n.s. not significant (4m.); unpaired two-tailed t-tests). **d**, Brain to body weight ratios, normalized to control littermates are normal at P0 and 4 month of age (*n*(P0, 4m.)= 6, 7 per genotype; n.s. not significant; unpaired two-tailed t-tests). Data is presented as mean ± SEM. Detailed statistics are provided in Supplementary Data 1.

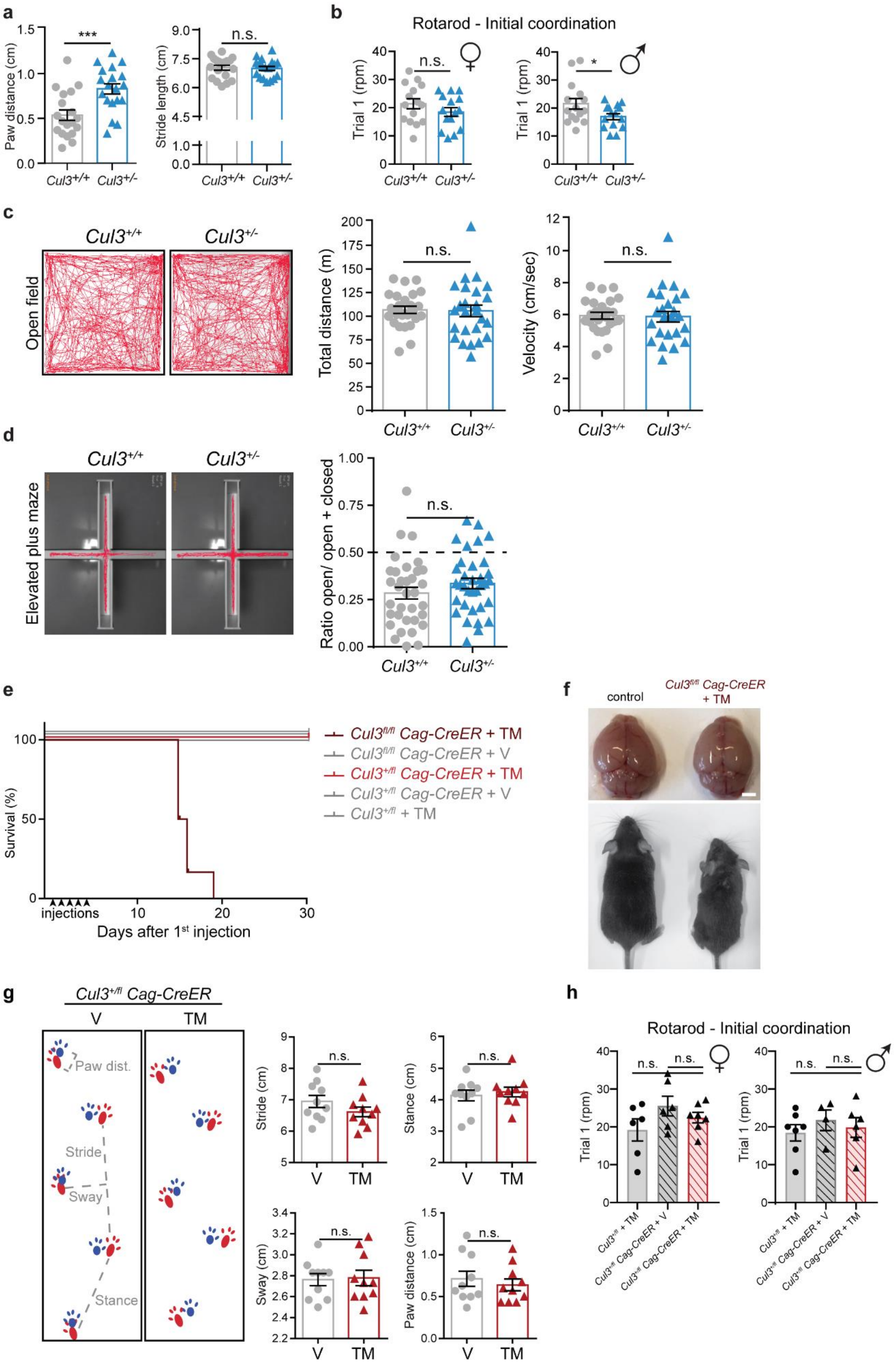

a
Paw distance (cm)
***
Stride length (cm)
n.s.
Cul3+/+
Cul3+/-
b
Rotarod - Initial coordination
Trial 1 (rpm)
n.s.
*
c
Open field
Total distance (m)
Velocity (cm/sec)
d
Elevated plus maze
Ratio open/ open + closed
e
Survival (%)
injections
Days after 1st injection
Cul3fl/fl Cag-CreER + TM
Cul3fl/fl Cag-CreER + V
Cul3+/fl Cag-CreER + TM
Cul3+/fl Cag-CreER + V
Cul3+/fl + TM
f
control
Cul3fl/fl Cag-CreER + TM
g
Cul3+/fl Cag-CreER
V
TM
Paw dist.
Stride
Sway
Stance
Stride (cm)
Stance (cm)
Sway (cm)
Paw distance (cm)
h
Rotarod - Initial coordination
Cul3+/fl + TM
Cul3+/fl Cag-CreER + V
Cul3+/fl Cag-CreER + TM

Supplementary Figure 2 Further behavioral features of *Cul3* haploinsufficient mice and conditional *Cul3* deletion in juvenile mice.

a, Increased paw distance (left), normal stride length (right) in *Cul3*$^{+/-}$ animals (*n*= 19 mice per genotype, littermates; ***P=0.001; n.s. not significant; two-tailed t-test). **b**, Accelerating RotaRod revealing defects in motor coordination in *Cul3*$^{+/-}$ male (b, right) but not in female (b, left) mice. Graph shows final rpm of trial 1 on day one, i.e: initial coordination (*n*= 15 female and male animals per genotype; *P=0.04; n.s. not significant; 2-way ANOVA and Sidak's multiple comparison test and unpaired two-tailed t-test). **c**, Normal exploratory behavior in the open field. Representative trajectories (left), no differences in total distance moved (center) and velocity (right) between *Cul3*$^{+/+}$ and *Cul3*$^{+/-}$ mice (*n*= 25 sex-matched littermate pairs; n.s. not significant; unpaired two-tailed t-tests). **d**, Trajectories of *Cul3*$^{+/-}$ mice in elevated plus maze (total duration 6 min) (left) and quantification of the ratio of time spent on open/ open+ closed arm (*n*= 34 sex-matched littermate pairs; n.s. not significant; unpaired two-tailed t-test). **e**, Mice with induced homozygous deletion of *Cul3* (*n(Cul3*$^{fl/fl}$ *Cag-CreER* + TM*)*= 6 mice) die within 20 days after first tamoxifen injection. Normal survival probability upon induced heterozygous deletion (*n(Cul3*$^{+/fl}$ *Cag-CreER* + TM*)*= 24) and controls (*n(Cul3*$^{+/fl}$ + TM*)*= 13, *n(Cul3*$^{+/fl}$ *Cag-CreER* + V*)*= 22, *n(Cul3*$^{fl/fl}$ *Cag-CreER* + V*)*= 2). **f**, *Cul3*$^{fl/fl}$ *Cag-CreER* + TM mouse and littermate control 15 days after first TM injection. **g,** Gait analysis of tamoxifen and vehicle treated *Cul3*$^{+/fl}$ *Cag-CreER* mice (representative foot-prints, left), normal stride, stance, sway or paw distance (*n*= 10 mice per condition; unpaired two-tailed t-tests). **h,** Accelerating RotaRod revealing normal motor coordination in treated and vehicle treated *Cul3*$^{+/fl}$ *Cag-CreER* mice, shown: final rpm on day one - trial 1 in females (h, left) and males (h, right) (*n(Cul3*$^{+/fl}$ + TM*)*= 7 males, 6 females, *n(Cul3*$^{+/fl}$ *Cag-CreER* + V*)*= 4 males, 6 females, *n(Cul3*$^{+/fl}$ *Cag-CreER* + TM*)*= 6 males, 7 females; n.s. not significant; 1-way ANOVA and Sidak's multiple comparison test and unpaired two-tailed t-test). Data presented as % survival (e), scatter plot with mean ± SEM (a, b, c, d, g, h). Scale bar: 2,5 mm (f, top). Detailed statistics are provided in Supplementary Data 1.

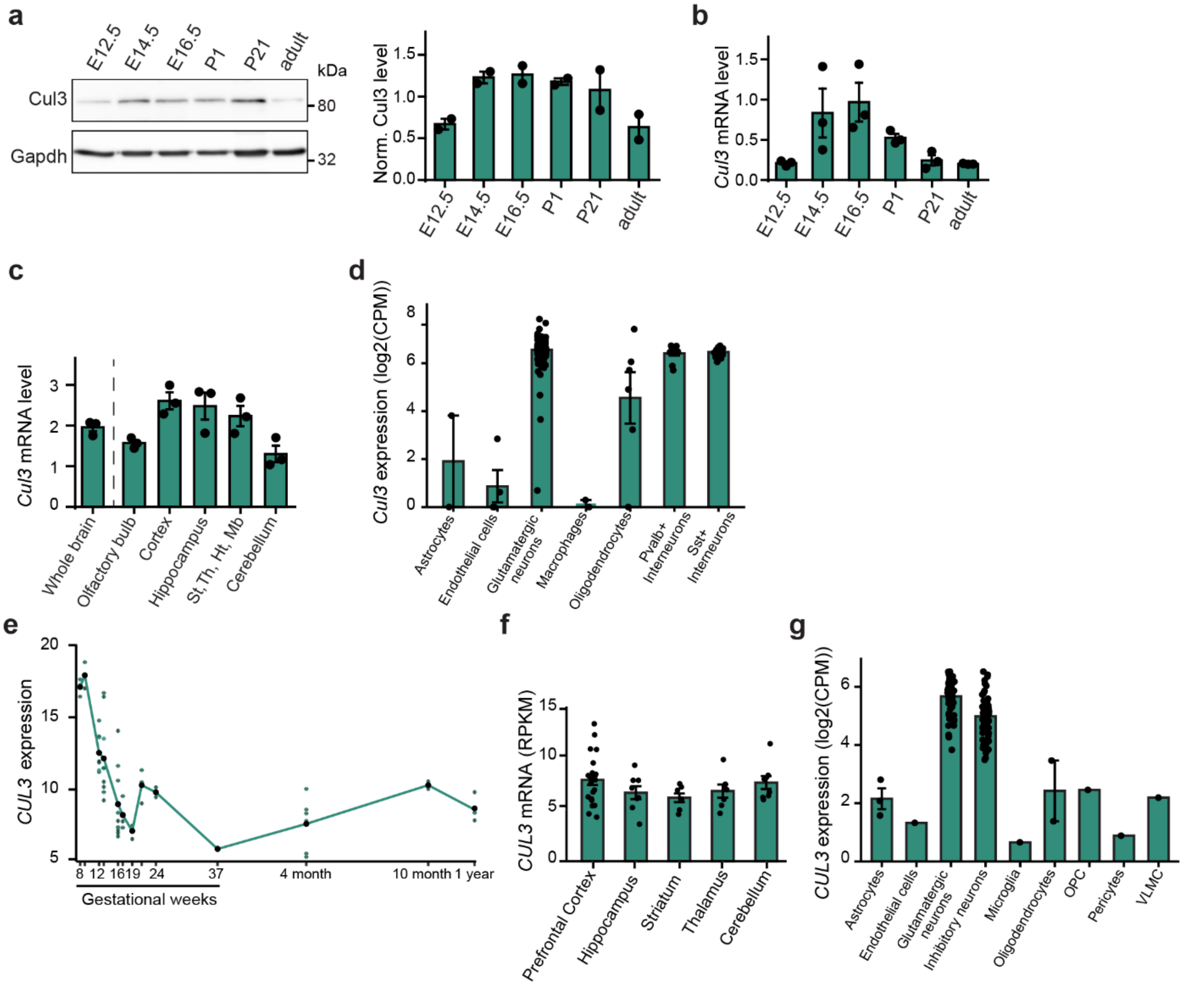

Supplementary Figure 3 ***Cul3*** **expression peaks during early development in both, mouse and humans.**

a, Western blot and quantification of E12.5, E14.5, E16.5, P1, P21 and adult brain lysates of C57BL/6J wild-type animals show highest Cul3 protein levels during developmental time-windows important for brain development (*n*(pooled tissue)= 3 animals per time point, *N*(WB)= 2). **b**-**c**, Quantitative real-time PCR analysis of *Cul3*, in brain development (b) and in adult brain regions (c) of C57BL/6J wild-type animals, confirms expression peaks during E14.5 and E16.5 and in cortex and hippocampal tissue. Lower *Cul3* levels were observed in the olfactory bulbs and the cerebellum (St= Striatum, Th= Thalamus, Ht= Hypothalamus, Mb= Midbrain) (*n*(tissue)= 3 animals, *N*(qPCR)= 3; ΔCq expression values are plotted). **d**, Normalized *Cul3* expression across cell types in the adult mouse brain based on data from the Allen Cell Types Database [© 2015 Allen Institute for Brain Science. Allen Cell Types Database. Available from https://portal.brain-map.org/atlases-and-data/rnaseq]. Data points indicate individual cell type clusters that were aggregated for this analysis. **e**, *CUL3* expression in cortical samples across human development based on data from the BrainSpan Atlas [© 2010 Allen Institute for Brain Science. BrainSpan Atlas of the Developing Human Brain. Available from http://www.brainspan.org/static/download.html]. X-axis shows age and y-axis shows expression in RPKM. Data points indicate individual samples. **f**, *CUL3* expression in RPKM across regions of the adult human brain based on data from the BrainSpan Atlas [© 2010 Allen Institute for Brain Science. BrainSpan Atlas of the Developing Human Brain. Available from

http://www.brainspan.org/static/download.html]. Data points indicate individual samples. **g**, Normalized *CUL3* expression across cell types in the adult human brain based on data from the Allen Cell Types Database [© 2015 Allen Institute for Brain Science. Allen Cell Types Database. Available from https://portal.brain-map.org/atlases-and-data/rnaseq]. Data points indicate individual cell type clusters that were aggregated for this analysis. Data is presented as mean ± SEM.

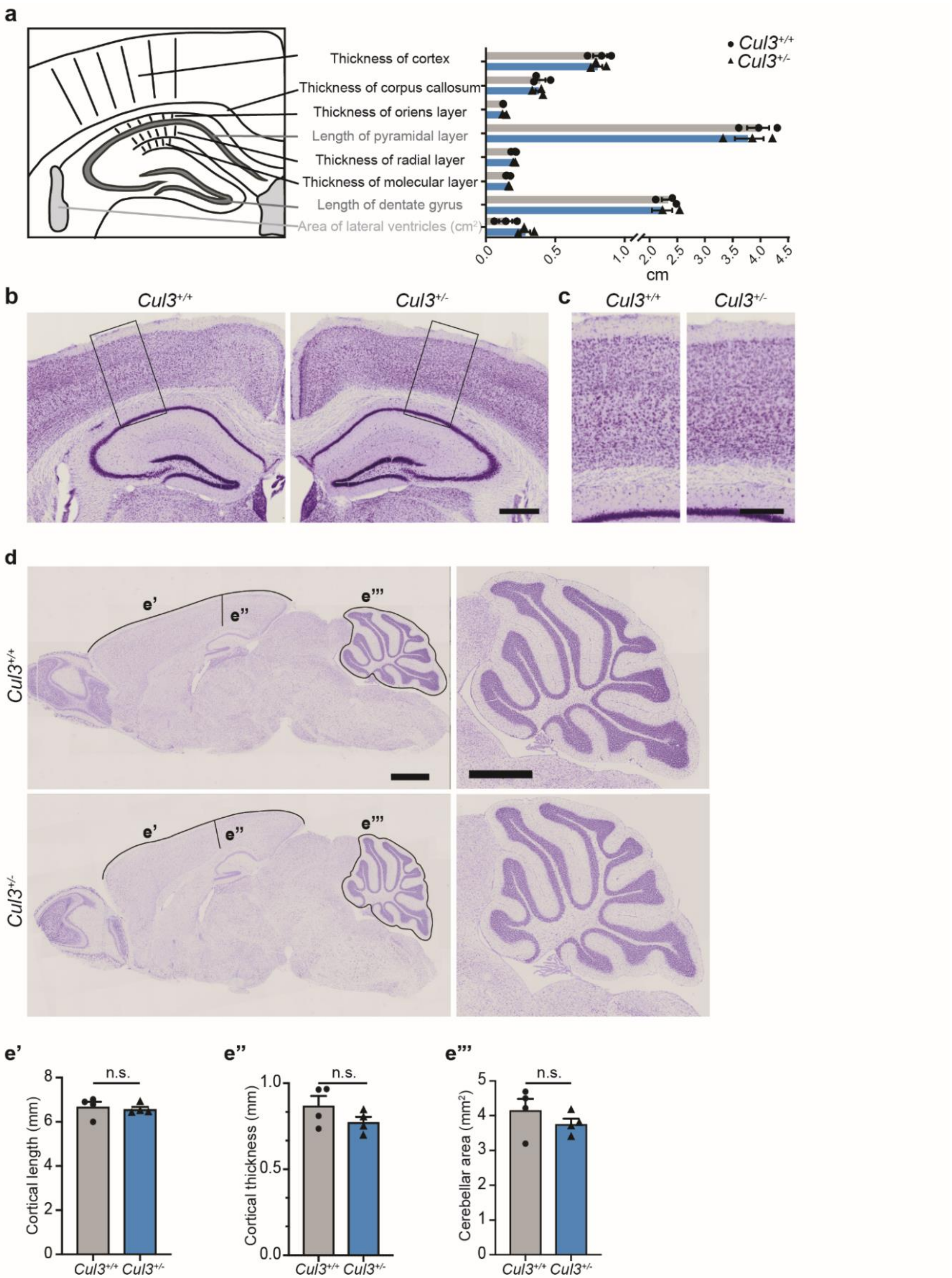

Supplementary Figure 4 Gross brain morphology of *Cul3* haploinsufficient mice appears normal.

a, Scheme of coronal forebrain sections and the measured brain features in adult *Cul3*$^{+/-}$ and *Cul3*$^{+/+}$ littermates revealed no differences between genotypes (*n*= 3 mice per genotype; unpaired two-tailed t-tests). **b**-**c**, Representative Nissl stainings of coronal forebrain sections in mutant and

wild-type mice, analyzed in (a) and close-ups of cortical columns in boxed regions in (c) (*n*= 3 mice per genotype). **d-e'''**, Overview (left) and cerebellar close-up (right) of Nissl stained adult sagittal brain sections and measurements of cortical length (e'), cortical thickness (e'') and cerebellar area (e''') confirmed these results, despite slight, yet not significant, decreases in the latter could be observed (*n*= 4 mice per genotype, littermates; n.s. not significant; unpaired two-tailed t-tests). Data is presented as mean ± SEM. Scale bars: 500 µm in (b), 250 µm in (c), 1 mm in (d, left and right). Detailed statistics are provided in Supplementary Data 1.

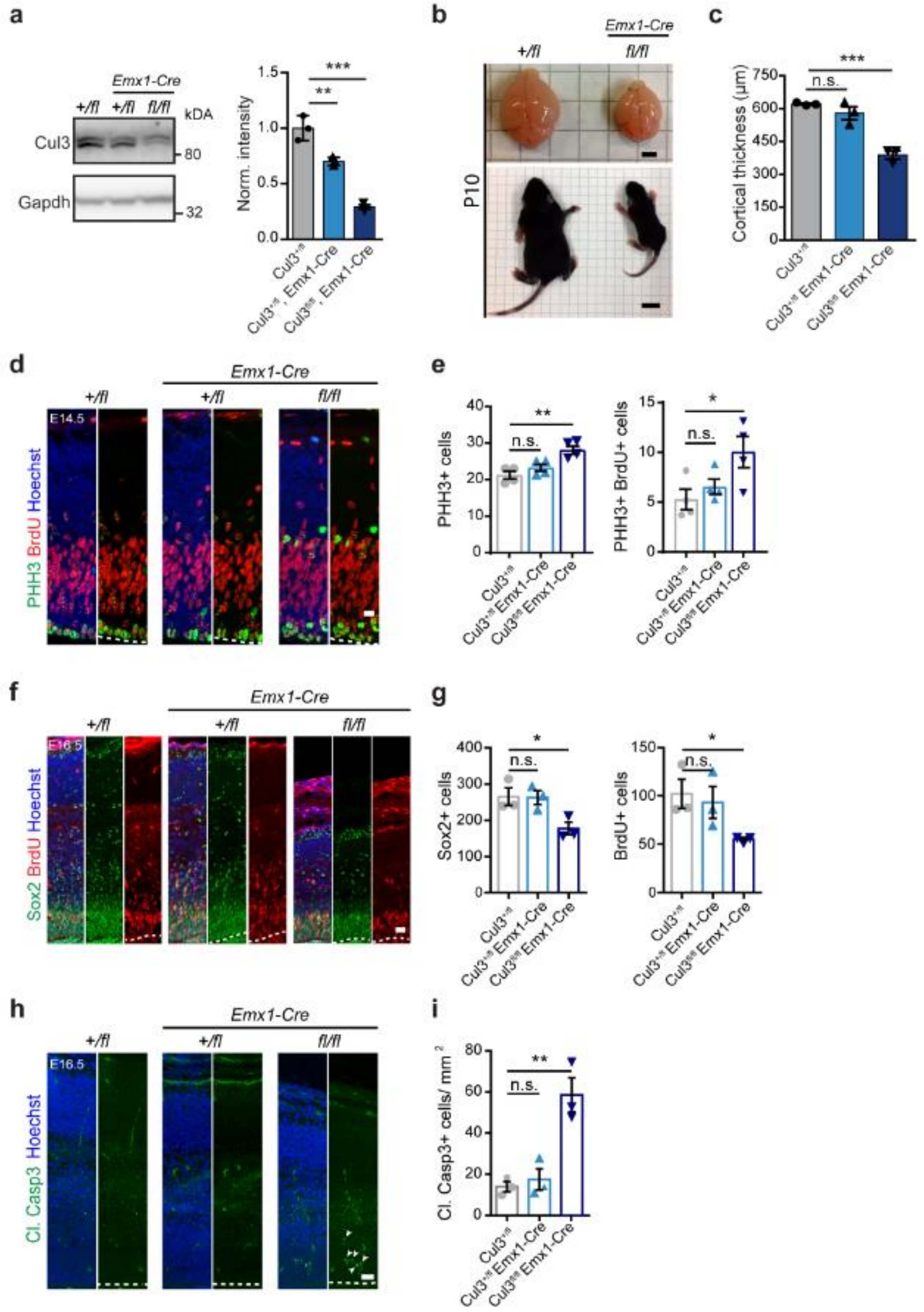

Supplementary Figure 5 *Cul3* loss leads to cell cycle defects and reduced neuronal survival in mice.

a, Western blot and quantification of *Cul3*$^{+/fl}$, *Cul3*$^{+/fl}$ *Emx1-Cre* and *Cul3*$^{fl/fl}$ *Emx1-Cre* E16.5 brain lysates shows a strong reduction of Cul3 levels in conditional homozygous embryos (*n*= 3 littermate embryos per genotype; **P=0.006, ***P<0.0001; 1-way ANOVA and Sidak's multiple comparisons test). **b**, Representative images of ten day-old *Cul3*$^{fl/fl}$ *Emx1-Cre* pups showing that

mutant animals are smaller than their *Cul3*$^{+/fl}$ control littermates. Hindbrain regions are comparable, forebrain structures are severely reduced in size in the conditional homozygous animals. **c**, Cortical thickness measured in Nissl stainings of coronal brain sections from *Cul3*$^{+/fl}$, *Cul3*$^{+/fl}$ *Emx1-Cre* and *Cul3*$^{fl/fl}$ *Emx1-Cre* newborn pups (P0), show severe cortical thinning in the latter (*n*= 3 pups per genotype; ***P=0.0005, n.s. not significant; 1-way ANOVA and Sidak's multiple comparisons test). **d**, Representative images of E14.5 coronal brain sections, stained for the M-Phase marker Phospho-Histone H3 (PHH3) and for BrdU incorporation (2 hours pulse) in *Cul3*$^{+/fl}$, *Cul3*$^{+/fl}$ *Emx1-Cre* and *Cul3*$^{fl/fl}$ *Emx1-Cre* embryos. **e,** Analysis of PHH3+ cells indicates similar cell proliferation in the *Cul3*$^{+/fl}$ and *Cul3*$^{+/fl}$ *Emx1-Cre* cortex and increased numbers of cells in M-Phase in *Cul3*$^{fl/fl}$ *Emx1-Cre* embryos (e, left) and increased numbers of PHH3-BrdU double-positive (PHH3+ BrdU+) cells (e, right) (*n*= 4 littermates per genotype; *P=0.036; **P=0.002, n.s. not significant; 1-way ANOVA and Sidak's multiple comparisons tests). **f**, Representative images of E16.5 coronal brain sections, stained for the radial glia marker Sox2 and for BrdU incorporation (2 hour pulse) in *Cul3*$^{+/fl}$, *Cul3*$^{+/fl}$ *Emx1-Cre* and *Cul3*$^{fl/fl}$ *Emx1-Cre* embryos. **g**, Quantification of Sox2+ cells and BrdU+ cells reveals decreased numbers of cycling radial glia cells in the *Cul3*$^{fl/fl}$ *Emx1-Cre* developing forebrain (*n*= 3 littermates per genotype; *P=0.046 (Sox2+), *P=0.044 (BrdU+); 1-way ANOVA and Sidak's multiple comparisons tests). **h-i,** Immunofluorescent staining for apoptotic marker cleaved Caspase-3 shows increased cell death in the *Cul3*$^{fl/fl}$ *Emx1-Cre* E16.5 cortex (arrowheads: cl. Casp3+ cells) (*n*= 3 littermates per genotype; **P=0.003, n.s. not significant; 1-way ANOVA and Sidak's multiple comparisons tests). Data presented as mean ± SEM. Scale bars: 2.5 mm in (b, top), 1 cm in (b, bottom), 12.5 µm in (d), 25 µm in (f, h). Detailed statistics are provided in Supplementary Data 1.

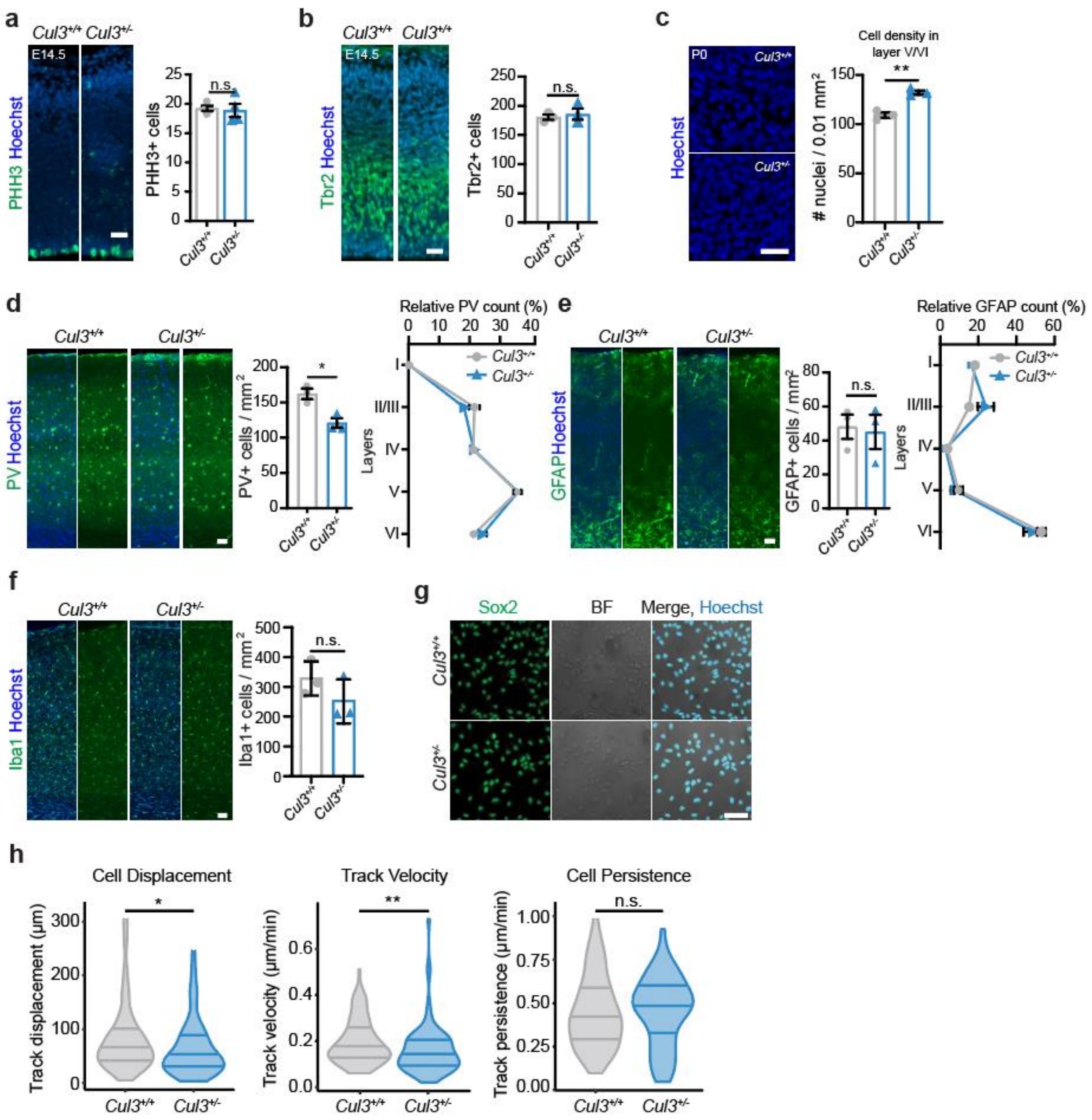

Supplementary Figure 6 Further histological characterization of *Cul3* haploinsufficient animals.

a, Representative images and quantification of E14.5 coronal brain sections, stained for the M-Phase marker Phospho-Histone H3 (PHH3) in *Cul3*$^{+/+}$ and *Cul3*$^{+/-}$ embryos showing comparable cell proliferation (*n*= 4 littermate pairs per genotype; n.s., not significant; unpaired two-tailed t-tests). **b,** Representative images and quantification of E14.5 coronal brain sections, stained for intermediate progenitors (IP) using Tbr2 indicates similar numbers of IP in the SVZ (*n*= 3 littermate pairs per genotype; n.s., not significant; unpaired two-tailed t-tests). **c,** Cell density analysis in cortical layers V/VI of P0 wild-type and heterozygous cortices reveals a significant increase in the number of cell nuclei, indicating an accumulation of a substantial number of neurons in lower layers (*n*= 3 littermate pairs per genotype; **P=0.002; unpaired two-tailed t-tests). **d-e**, Representative images and quantification of immunofluorescent stainings against PV+

interneurons and GFAP+ astrocytes in adult coronal cortical sections, showing reduced numbers of PV+ cells (d) but not astrocytes (e) in mutant animals. Analysis of relative numbers of PV+ (d, right) and GFAP+ (e, right) cells showed no difference in layer distribution. (*n*= 3 littermate pairs per genotype; *P=0.013, n.s., not significant; unpaired two-tailed t-tests). **f,** No difference in the number of Iba1+ microglia in the adult *Cul3*$^{+/-}$ cortex (*n*= 3 per genotype; n.s. not significant; unpaired two-tailed t-test). **g,** Representative pictures of *Cul3*$^{+/+}$ and *Cul3*$^{+/-}$ NPC preparations stained for Sox2. **h** Cell tracks of NPCs detaching from neurosphere into embedding bovine collagen matrix imaged in a single plane. Cell displacement, cell velocity and migratory persistence were quantified in the imaging plane and compared between *Cul3*$^{+/+}$ and *Cul3*$^{+/-}$ cells (*n*(spheres)= 3 per genotype, *n*(cells)= 30 per replicate; *P=0.019; **P=0.002; Wilcoxon rank sum test). Data presented as mean ± SEM or violin plots with median and first and third quartiles. Scale bars: 50 μm in (a, b, d, e, f, g), 25 μm in (c). Detailed statistics are provided in Supplementary Data 1.

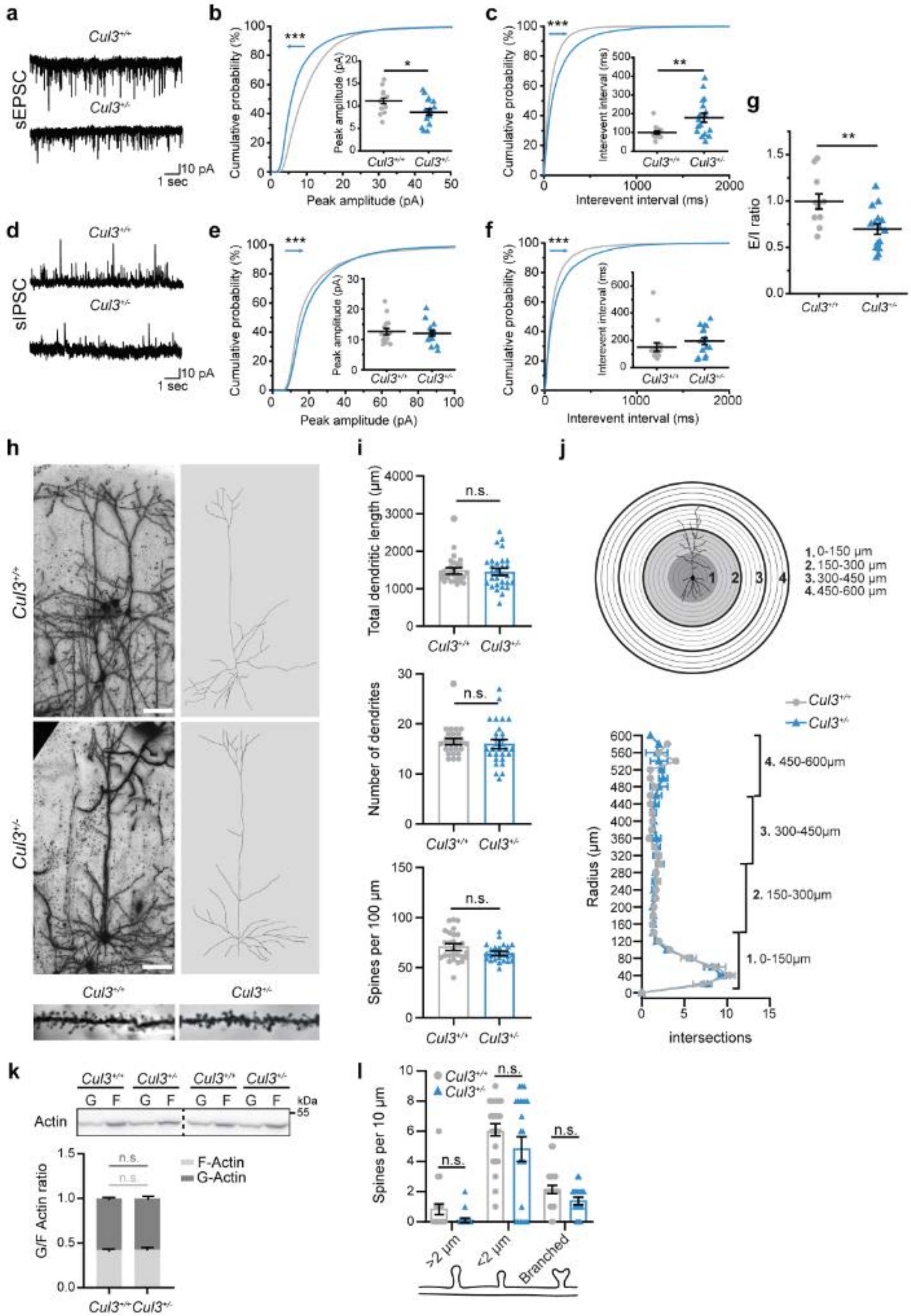

a
sEPSC
Cul3+/+
Cul3+/-
10 pA
1 sec
b
Cumulative probability (%)
Peak amplitude (pA)
***
*
c
Interevent interval (ms)
**
d
sIPSC
e
f
g
E/I ratio
h
i
Total dendritic length (µm)
Number of dendrites
Spines per 100 µm
n.s.
j
1. 0-150 µm
2. 150-300 µm
3. 300-450 µm
4. 450-600 µm
Radius (µm)
intersections
k
Actin
G
F
kDa
55
G/F Actin ratio
F-Actin
G-Actin
l
Spines per 10 µm
>2 µm
<2 µm
Branched

Supplementary Figure 7 Reduced circuit activity in layer 2/3 pyramidal neurons of adult *Cul3*$^{+/-}$ mice, but normal neuronal morphology.

a,**d**, Representative sEPSC (a) and sIPSC (d) traces, recorded by whole cell patch-clamp, at holding potential -70 mV (sEPSC) and +10mV (sIPSC), from layer 2/3 neurons in somatosensory cortex. **b**, Cumulative probability distribution and quantification of sEPSC (b) and sIPSC (E) amplitudes (*P=0.016, ***P<0.0003; Kolmogorov-Smirnov test). **c**, Cumulative probability distribution and quantification of sEPSC interevent intervals (IEI) (*n*(*Cul3*$^{+/+}$)= 15 cells, *n*(*Cul3*$^{+/-}$)= 17 cells from 6 mice respectively; **P=0.005, ***P<0.0001; unpaired two-tailed t-test, Kolmogorov-Smirnov test). **f**, Cumulative probability distribution and quantification of mean sIPSC IEI; (*n*(*Cul3*$^{+/+}$)= 16 cells, *n*(*Cul3*$^{+/-}$)= 16 cells from 6 mice; ***P<0.0001; Kolmogorov-Smirnov test). **g**, Calculation of the excitation/inhibition (E/I) ratio reveals a reduced E/I balance (n(*Cul3*$^{+/+}$)= 11 cells, n(*Cul3*$^{+/-}$)= 15 cells from 6 mice; **P=0.005; unpaired two-tailed t-test). **h**-**j**, Golgi staining and analysis of morphology and spine density of layer 2/3 pyramidal neurons in the somatosensory cortex. Brightfield images (h, left) and Imaris reconstructions (h, right) of *Cul3*$^{+/+}$ and *Cul3*$^{+/-}$ neurons, close-ups of dendrites with spines (h, bottom). Quantification did not reveal any differences in total dendritic length (i, top), number of dendrites (i, center) or spine density (i, bottom) (*n*(cells)= 27-29 from 3 mice per genotype); Sholl analysis was comparable between the mutant and wild-type (j, top: scheme; j, bottom: quantification); (*n*(mice)= 3 mice per genotype, at least 9 cells per animal; n.s. not significant; unpaired two-tailed t-tests or two-tailed Mann-Whitney U-test). Analyses were done in adult littermate male mice. **k**, Western Blot (top) and analysis of globular (G-) and filamentous (F-) actin indicate that the rate of actin polymerization is comparable between wild-type and *Cul3*$^{+/-}$ lysates (*n*= 3 per genotype; n.s. not significant; 2-way ANOVA and Sidak's multiple comparison test). **l**, Quantification of spine morphology did not reveal any differences (*n*(cells)= 21-24 from 3 mice per genotype). Data is shown as mean ± SEM and scatter plots in (b, c, e, f, g, i, l) and as stacked bar-plots of mean ± SEM in (k). Data presented as connected mean ± SEM in (j). Scale bars: 50 µm in (h, top) and 5 µm in (h, bottom). Detailed statistics are provided in Supplementary Data 1.

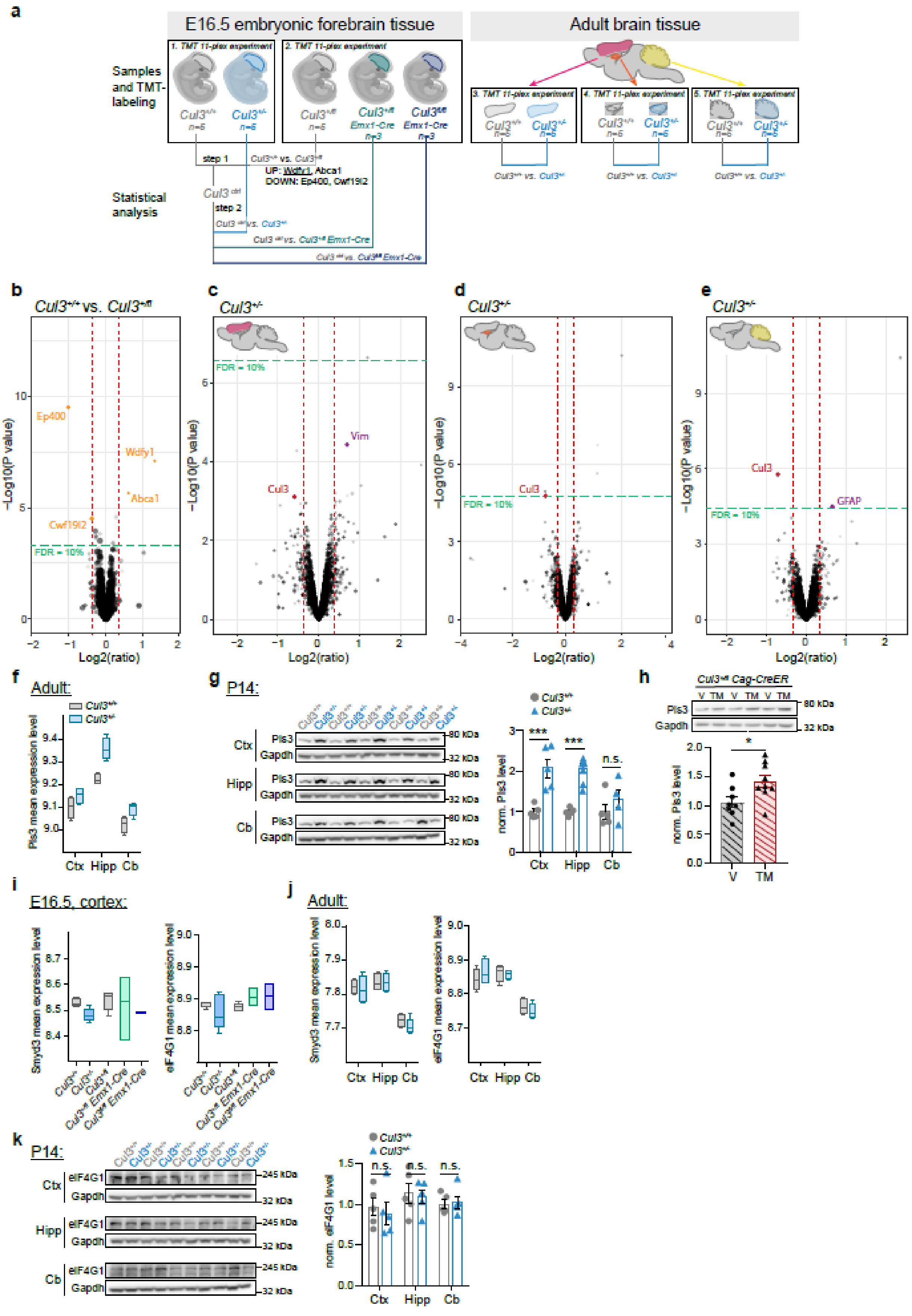
a
E16.5 embryonic forebrain tissue
Adult brain tissue
Samples and TMT-labeling
Statistical analysis
step 1
step 2
UP: Wdfy1, Abca1
DOWN: Ep400, Cwf19l2
b
c
d
e
FDR = 10%
Ep400
Wdfy1
Abca1
Cwf19l2
Vim
Cul3
GFAP
−Log10(P value)
Log2(ratio)
f
Adult:
Pls3 mean expression level
Ctx
Hipp
Cb
g
P14:
Pls3
Gapdh
80 kDa
32 kDa
norm. Pls3 level
n.s.
h
V
TM
i
E16.5, cortex:
Smyd3 mean expression level
eIF4G1 mean expression level
j
Adult:
k
P14:
eIF4G1
245 kDa
norm. eIF4G1 level

Supplementary Figure 8 Minor protein composition alterations in the adult *Cul3*$^{+/-}$ brain.
a, Experimental design of proteomic analyses in embryonic forebrain and adult brain tissue. In total 5 time 11-plex TMT experiments were performed. **b,** Volcano plot of deregulated proteins at 10% FDR between embryonic control samples (i.e. *Cul3*$^{+/+}$ vs. *Cul3*$^{+/fl}$) reveals only four differentially expressed proteins, i.e. Cwf19l2, Ep400, Abca1 and Wdfy1. **c,** Volcano plot of deregulated proteins at 10% FDR cut-off in the adult *Cul3*$^{+/-}$ cortex (details in Supplementary Data 8). **d**, Volcano plot of deregulated proteins at 10% FDR cut-off in the adult *Cul3*$^{+/-}$ hippocampus with 3 up- and 1 down-regulated protein (details in Supplementary Data 8). **e**, Volcano plot of deregulated proteins at 10% FDR cut-off in the adult *Cul3*$^{+/-}$ cerebellum with 2 up- and 1 down-regulated proteins (details in Supplementary Data 8). Purple: Cytoskeletal proteins, red: Cul3 (c-e). **f**, Raw protein expression levels indicate mildly elevated Pls3 levels in all analyzed adult *Cul3*$^{+/-}$ brain regions. **g**, Western blot (e, left) and analysis (e, right) of Gapdh* normalized intensities of Pls3 indicate an increase in all analyzed *Cul3*$^{+/-}$ juvenile (P14) brain regions (*n*= 5 per genotype; ***P=0.0002, n.s. not significant; 2-way ANOVA and Sidak's multiple comparisons test). **h**, Pls3 protein levels also increase upon tamoxifen-induced reduction of *Cul3* expression (n(*Cul3*$^{+/fl}$ *Cag-CreER* + V)= 8 mice and n(*Cul3*$^{+/fl}$ *Cag-CreER + TM*)= 9 mice; *P=0.024; unpaired two-tailed t-test). **i**, Analysis of raw expression levels of Smyd3 and eIF4G1 in E16.5 embryonic cortical proteomic datasets did not show any changes in protein abundance. **j**, Raw expression levels of Smyd3 and eIF4G1 proteins were unchanged in all analyzed adult *Cul3*$^{+/-}$ brain regions. **k**, Western blot (left) and analysis of Gapdh* normalized intensities (right) show no differences of Pls3 levels in any of the analyzed juvenile (P14) *Cul3*$^{+/-}$ brain regions (*n*= 5 per genotype; n.s. not significant; 2-way ANOVA and Sidak's multiple comparisons test). *Note that Gapdh loading control in panels (g) and (k) are the same - membrane was cut to analyze both, Pls3 and eIF4G1. Boxplot shows median value and 25–75th percentile, whiskers show the minimum and the maximum in (f), (i-j). Detailed statistics are provided in Supplementary Data 1.

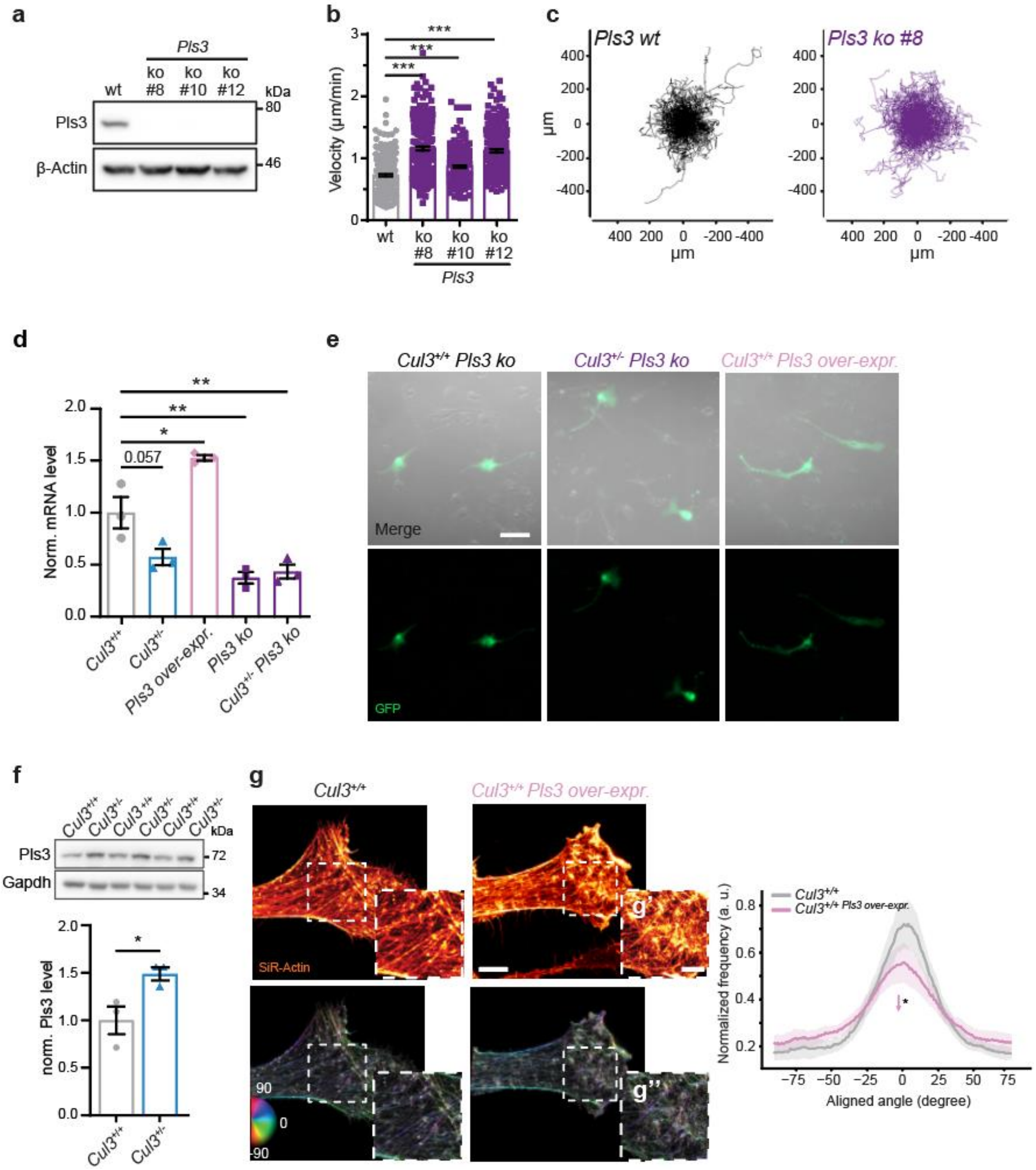

Supplementary Figure 9 Pls3 regulates cell migration dynamics and actin cytoskeleton organization in *Cul3* haploinsufficient NPCs.

a, Representative western blot of B16-F1 wild-type and CRISPR/Cas9 knockout cell clones confirm lack of detectable Pls3 protein in 3 independently selected *Pls3* ko clones (labeled as ko#8, #10 and #12). **b**, 2D random migration assay was performed over a period of 15 hours in B16-F1 wild-type and *Pls3* ko cells. Average migration velocity for all cell tracks per cell type was plotted on the y-axis (µm/min). Results indicate significant increase in migration velocity in all *Pls3* knockout clones, relative to the B16-F1 wild-type (*n*(*wt*)= 237, *n*(*ko #8*)= 205, *n*(*ko #10*)= 222 and

n(*ko #12*)= 212 cells respectively; ***P<0.0001; Kuskal-Wallis test and Dunn's multiple correction test). **c**, Representative cell paths/trajectories of B16-F1 wild-type (black) and *Pls3 ko #8* cells (purple), extracted from data presented in panel b, indicate larger migration area upon *Pls3* knockout. **d,** Quantitative real-time PCR analysis of *Pls3* mRNA in *Cul3*$^{+/+}$ and *Cul3*$^{+/-}$ NPCs as well as of FACS sorted *Pls3 over-expressing* and *Pls3 loss of function* NPCs normalized to wild-type levels (ΔCq expression values; *n*= 3 per genotype; *P=0.015; **P<0.01; 1-way ANOVA and Sidak's multiple comparisons tests). **e,** Representative images of *Pls3 ko, Cul3*$^{+/-}$ *Pls3 ko and Pls3 over-expressing* NPC preparations, transfected cells (bright field), eGFP expression (successfully transfected cells). **f,** Western blot (top) and analysis (bottom) of Gapdh-normalized intensities of Pls3 indicate an increase in *Cul3*$^{+/-}$ NPCs (*n*= 3 per genotype; *P=0.03; unpaired two-tailed t-test). **g,** *Cul3*$^{+/+}$ and *Pls3 over-expressing* NPCs stained using SiR-actin. Cell protrusions were imaged employing STED-microscopy (close-up images in insets g', g''). Orientation distributions for actin (g, right) aligned to the dominant average orientation angle of microtubules are shown for *Cul3*$^{+/+}$ and *Cul3*$^{+/+}$ *Pls3 over-expressing* cells (*n*(*Cul3*$^{+/-}$)= 31 cells, (n(*Cul3*$^{+/+}$ *Pls3 over-expr.*)= 30 cells from three independent NPC preparations; *P=0.015; two-tailed Welch's t-test). Plotted is the average angle distribution per group ± 95% confidence intervals. Scale bars: 50 µm in (e), 5 µm in (g), 2.5 µm in (inset g'). Detailed statistics are provided in Supplementary Table 1. Data presented as mean ± SEM and scatter plots in (b, d, f). Detailed statistics are provided in Supplementary Data 1.

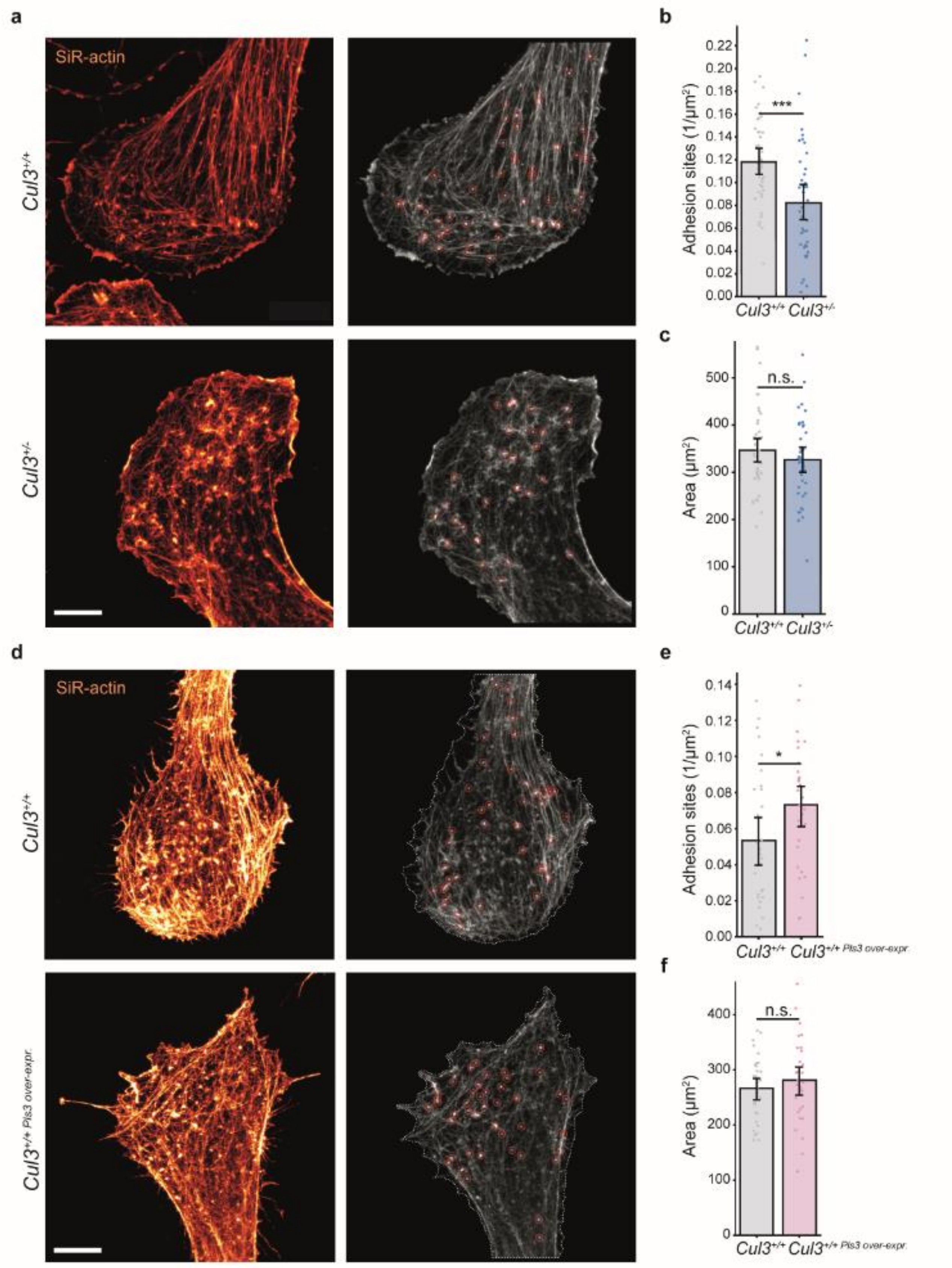

Supplementary Figure 10 Decreased number of focal adhesions in *Cul3*$^{+/-}$ NPCs.

a,**d** NPCs cultured on Poly-L-ornithine/Laminin were stained using SiR-actin (a, d) and cell protrusions were imaged employing STED-microscopy. **b,e**, The number of bright SiR-actin puncta (red circles in a, right and d, right), putative focal adhesion sites, are reduced in *Cul3*$^{+/-}$ NPCs and increased in *Pls3 over-expressing* NPCs. Puncta were counted and normalized to the area. (*n*(*Cul3*$^{+/+}$ and *Cul3*$^{+/-}$)= 43 per genotype from three independent NPCs preparations; (n(*Cul3*$^{+/+}$ and *Cul3*$^{+/+}$ *Pls3 over-expr.*)= 30 cells from three independent NPCs preparations; *P=0.03; ***P=0.0004; two-tailed Welch's t-test). **c,f**, Areas were comparable between wild-type and mutant as well as in *Pls3 over-expr.* cells (*n*(*Cul3*$^{+/+}$ and *Cul3*$^{+/-}$)= 43 per genotype from three independent NPCs preparations; (n(*Cul3*$^{+/+}$ and *Cul3*$^{+/+}$ *Pls3 over-expr.*)= 30 cells per genotype from three independent NPCs preparations; n.s. not significant, two-tailed Welch's t-test). Data presented as mean ± SEM and scatter plots. Scale bar: 5 µm in (a, d). Detailed statistics are provided in Supplementary Data 1.

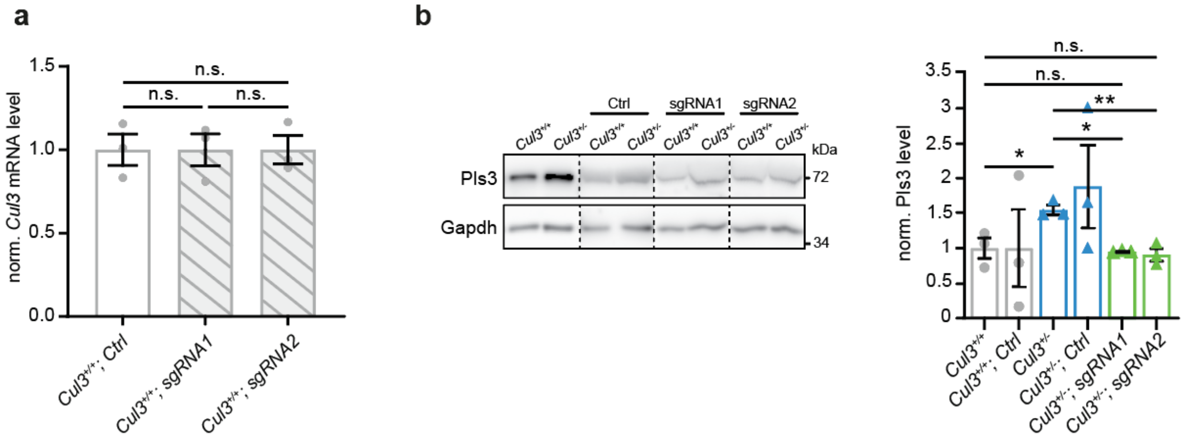

Supplementary Figure 11 Control guides and *Cul3*-targeting guides show similar *Cul3* expression levels.

a, Quantitative real-time PCR analysis of *Cul3* mRNA levels normalized to wild-type levels of transfected *Cul3*$^{+/+}$ mNPCs using their respective guide targeting the *Cul3* promoter. Expression of *Cul3* mRNA does not increase upon transfection with a control guide or *Cul3*-targeting guides. (ΔCq expression values; *n*= 3 replicates per condition; n.s. not significant; 1-way ANOVA and Sidak's multiple comparisons tests). **b,** Western blot (left) and analysis (right) of Gapdh-normalized intensities of Pls3 in *Cul3*$^{+/-}$ NPCs and FACS sorted NPCs using a control guide (Ctrl sgRNA) and two independent sgRNAs. Levels of Pls3 indicate an increase in *Cul3*$^{+/-}$ and *Cul3*$^{+/-, Ctrl}$ NPCs, while Pls3 is comparable to wild-type levels in NPCs expressing two different sgRNAs (*n*= 3 per genotype; *P=0.01; **P=0.007; n.s. not significant; 1-way ANOVA and Sidak's multiple comparisons tests). Data presented as mean ± SEM and scatter plots. Detailed statistics are provided in Supplementary Data 1.

Supplementary Table 1 Primer sequences used.

Name	Sequence	Primer purpose
Cul3 knockout allele fwd	GGAAACCTAAAGTTTTTATGCATG	Cul3 mouse knockout allele validation
Cul3 knockout allele rev	TTTGTCTGGACCAAATATGGCAGCCCAAACC	Cul3 mouse knockout allele validation
Cul3 fwd qPCR primer	AAGGTGGTGGAGAGGGAACT	Cul3 qPCR primer forward
Cul3 rev qPCR primer	TCAAACCATTTGGCACACGAC	Cul3 qPCR primer reverse
Cul3 fwd qPCR primer 2	CAGCGGGTCCTCACAAAAGA	Cul3 qPCR primer forward
Cul3 rev qPCR primer 2	CTGGGTCGGATTCACCTTGT	Cul3 qPCR primer reverse
Pls3 fwd qPCR primer	TCTAGAAGGGGAAACTCGGG	Pls3 qPCR primer forward
Pls3 rev qPCR primer	GGATCACCAGAGCATCCTGC	Pls3 qPCR primer reverse
Pls1 fwd qPCR primer	CCATGCCTACACAAGCCTGA	Pls1 qPCR primer forward
Pls1 rev qPCR primer	GCGTCTGCAAGGTCACTGTA	Pls1 qPCR primer reverse
INA fwd qPCR primer	CCAGGCACGTACCATTGAGAT	INA qPCR primer forward
INA rev qPCR primer	CAATGCTGTCCTGGTAGCCG	INA qPCR primer reverse
Pgk1 fwd qPCR primer	AAAGTCAGCCATGTGAGCACT	Pgk1 qPCR primer forward
Pgk1 rev qPCR primer	ACTTAGGAGCACAGGAACCAAA	Pgk1 qPCR primer reverse
Gapdh fwd qPCR primer	AACGGGAAGCTCACTGGCAT	Gapdh qPCR primer forward
Gapdh rev qPCR primer	GCTTCACCACCTTCTTGATG	Gapdh qPCR primer reverse
Pls3 knockout allele cloning primer fwd	CACCGTATCGCTAAAACCTTCCGAA	Pls3 knockout cloning primer
Pls3 knockout allele cloning primer rev	AAACTTCGGAAGGTTTTAGCGATAC	Pls3 knockout cloning primer
Pls3 knockout allele fwd	ATGCAGCACTGAATGTGTTTGG	Pls3 knockout allele validation
Pls3 knockout allele rev	ACCAATATGTGACCCAAGACCC	Pls3 knockout allele validation
Pls3 knockout allele sequencing primer fwd	CAGGAAACAGCTATGAC	Pls3 knockout allele cloning validation
Pls3-FW-XhoI-EGFPC1	ATATCTCGAGATATGGATGAGATGGCGACC	Pls3 overexpression cloning primer
Pls3-Rev-SalI-EGFPC1	TTTGTCGACTTACACTCTCTTCATCCC	Pls3 overexpression cloning primer
mCul3-prom_g1_top	ACCGCGAGACGAGATCCGGCCCA	Cul3 CRISPRa_guide 1 cloning primer forward
mCul3-prom_g1_bottom	AACTGGGCCGGATCTCGTCTCGC	Cul3 CRISPRa_guide 1 cloning primer reverse
mCul3-prom_g2_top	ACCGTCCCCCCTCCACCCTCTGGG	Cul3 CRISPRa_guide 2 cloning primer forward
mCul3-prom_g2_bottom	AACCCCAGAGGGTGGAGGGGGGAC	Cul3 CRISPRa_guide 2 cloning primer reverse
mCul3-prom_sequencing primer fwd	ACTATCATATGCTTACCGTAAC	Cul3 CRISPRa cloning validation
