# Supplementary material for: Cul3 regulates cytoskeleton protein homeostasis and cell migration during a critical window of brain development
Source: Nat Commun. 2021 May 24;12:3058. doi: 10.1038/s41467-021-23123-x (PMC8144225; doi:10.1038/s41467-021-23123-x)
Supplement: Supplementary file 3 — Description of Additional Supplementary Files [file 41467_2021_23123_MOESM3_ESM.pdf]

Description of Additional Supplementary Files

File Name: Supplementary Data 1.

Description: Detailed statistical analysis. Table displays detailed statistical analysis performed.

File Name: Supplementary Data 2.

Description: Detailed analysis of the Olfaction Habituation and Dishabituation (ODHD) test. Data displays detailed statistics derived from the ODHD test performed with *Cul3*$^{+/-}$ and *Cul3*$^{+/+}$ littermate animals (*n*= 24 mice per genotype; analyzed using 2-way ANOVA and Sidak's multiple comparison test; data related to Fig. 1g) as well as with *Cul3*$^{+/fl}$ + TM, *Cul3*$^{+/fl}$ *Cag-CreER* + V and *Cul3*$^{+/fl}$ *Cag-CreER* + TM mice (*n(Cul3*$^{+/fl}$ + TM*)*= 13, *n(Cul3*$^{+/fl}$ *Cag-CreER* + V*)*= 8, *n(Cul3*$^{+/fl}$ *Cag-CreER* + TM*)*= 10; littermate animals, analyzed using 2-way ANOVA and Sidak's multiple comparison test; data related to Fig. 2h).

File Name: Supplementary Data 3.

Description: Whole proteome analysis of deregulated proteins in the developing *Cul3*$^{+/-}$ brain. Table displays proteomic changes detected in the *Cul3+/-* developing cortex at E16.5 (*n*(*Cul3+/-*)= 5 per genotype; male mutant and control littermate pairs), including F-test performed, P-value significance thresholds were calculated for a false discovery rate level of 10-20%. Down-regulated proteins are depicted in green, up-regulated proteins are depicted in blue (data related to Fig. 5b).

File Name: Supplementary Data 4.

Description: Whole proteome analysis of deregulated proteins in *Cul3*$^{+/fl}$ *Emx1-Cre* developing forebrain. Table displays proteomic changes detected in the *Cul3*$^{+/fl}$ *Emx1-Cre* developing cortex at E16.5 (*n*(*Cul3*$^{+/fl}$ *Emx1-Cre)*= 3 per genotype; male mutant and control littermate pairs), including F-test performed, P-value significance thresholds were calculated for a false discovery rate level of 10-20%. Down-regulated proteins are depicted in green, up-regulated proteins are depicted in blue (data related to Fig. 5c).

File Name: Supplementary Data 5.

Description: Whole proteome analysis of deregulated proteins in *Cul3*$^{fl/fl}$ *Emx1-Cre* developing forebrain. Table displays proteomic changes detected in the *Cul3*$^{fl/fl}$ *Emx1-Cre* developing cortex at E16.5 (*n*(*Cul3*$^{+/fl}$ *Emx1-Cre)*= 3 per genotype; male mutant and control littermate pairs), including F-test performed, P-value significance thresholds were calculated for a false discovery rate level of 10-20%. Down-regulated proteins are depicted in green, up-regulated proteins are depicted in blue (data related to Fig. 5d).

File Name: Supplementary Data 6.

Description: Functional annotation analysis of deregulated proteins in *Cul3* mutant embryonic forebrain tissue. Gene ontology analysis (DAVID 6.8 functional annotation) of differently expressed genes at 20% FDR of the *Cul3$^{fl/fl}$ Emx1-Cre* forebrain identified down- and up- regulated proteins to be involved in regulating activity of RNA polymerase II, the proteasome core complex, neurogenesis and actin and microtubule cytoskeletal organization. Significant GO-terms in red: RNA polymerase II core complex, GO: 0005665, adj. p-value= 0.002; proteasome core complex GO: 0005839, adj. p-value= 4.9e-07 (data related to Fig. 5e).

File Name: Supplementary Data 7.

Description: Overlap of up- and down-regulated proteins in *Cul3* mutant embryonic cortex. Comparison of ratios of up- and down-regulated proteins in the *Cul3$^{+/-}$, Cul3$^{+/fl}$ Emx1-Cre* and *Cul3$^{fl/fl}$ Emx1-Cre* cortex at log 0.1 change. Overlapping down-regulated proteins are highlighted in green, up-regulated proteins are highlighted in blue (data related to Fig. 5f, g). Fitting the mean raw expression levels of Pls3 and INA to a linear regression model indicates that these proteins follow a genotype-dependent dose response (Pls3*:* R^2 = 0.999; INA*:* R^2 = 0.932; *Cul3$^{+/fl}$* < *Cul3$^{+/fl}$ Emx1-Cre* < *Cul3$^{fl/fl}$ Emx1-Cre*; data related to Fig. 5h).

File Name: Supplementary Data 8.

Description: Whole proteome analysis of deregulated proteins in the adult *Cul3$^{+/-}$* brain. Table displays proteomic changes detected in the adult *Cul3$^{+/-}$* cortex, hippocampus and cerebellum (*n*(cortex and hippocampus: 4 littermates per genotype); (cerebellum: 5 littermates per genotype)), including F-test performed, P-value significance thresholds were calculated for a false discovery rate level of 10-20%. Down-regulated proteins are depicted in green, up-regulated proteins are depicted in blue (data related to Supplementary Fig. 8c-e).

File Name: Supplementary Data 9.

Description: Experimental design of proteomic analysis in adult *Cul3* brain tissue. Digested protein samples were labeled with Tandem Mass Tag (TMT) 11-plex reagents. For the adult dataset, where the number of samples exceeded the number of available TMT channel, one combined TMT sample was generated for each tissue. For adult and embryonic analysis, in total 5 time 11-plex TMT experiments were performed (data related to Supplementary Fig. 8a).

File Name: Supplementary Data 10.

Description: Experimental design of proteomic analysis in embryonic *Cul3* forebrain tissue. Digested protein samples were labeled with Tandem Mass Tag (TMT) 11-plex reagents. For the embryonic

dataset, one TMT sample contained *Cul3*$^{+/+}$ and *Cul3*$^{+/-}$ samples, while the other sample contained the *Cul3*$^{+/fl}$*, Cul3*$^{+/fl}$ *Emx1-Cre* and *Cul3*$^{fl/fl}$ *Emx1-Cre* samples*.* For adult and embryonic analysis, in total 5 time 11-plex TMT experiments were performed (data related to Supplementary Fig. 8a).

File Name: Supplementary Movie 1.

Description: *In vitro* migration assay of matrigel embedded neurospheres generated from *Cul3*$^{+/+}$ NPCs. Representative bright-field movie of an *in vitro* migration assay of a matrigel embedded neurosphere generated from *Cul3*$^{+/+}$ NPCs. Migratory abilities were tracked over a time-course of 72 hours after plating.

File Name: Supplementary Movie 2.

Description: *In vitro* migration assay of matrigel embedded neurospheres generated from *Cul3*$^{+/-}$ NPCs. Representative bright-field movie displays an *in vitro* migration assay of a matrigel embedded neurosphere generated from *Cul3*$^{+/-}$ NPCs. Migratory abilities were tracked over a time-course of 72 hours after plating, revealing that mutant NPCs move less and have reduced migration speed.
