# Supplementary material for: Cul3 regulates cytoskeleton protein homeostasis and cell migration during a critical window of brain development
Source: Nat Commun. 2021 May 24;12:3058. doi: 10.1038/s41467-021-23123-x (PMC8144225; doi:10.1038/s41467-021-23123-x)
Supplement: Supplementary file 16 — Reporting Summary [file 41467_2021_23123_MOESM16_ESM.pdf]

nature research

Corresponding author(s): Gaia Novarino

Last updated by author(s): Mar 12, 2021

Reporting Summary

Nature Research wishes to improve the reproducibility of the work that we publish. This form provides structure for consistency and transparency in reporting. For further information on Nature Research policies, seeAuthors & Referees and theEditorial Policy Checklist .

Statistics

For all statistical analyses, confirm that the following items are present in the figure legend, table legend, main text, or Methods section.

n/a	Confirmed	
☐	☒	The exact sample size (n) for each experimental group/condition, given as a discrete number and unit of measurement
☐	☒	A statement on whether measurements were taken from distinct samples or whether the same sample was measured repeatedly
☐	☒	The statistical test(s) used AND whether they are one- or two-sided<br>*Only common tests should be described solely by name; describe more complex techniques in the Methods section.*
☒	☐	A description of all covariates tested
☐	☒	A description of any assumptions or corrections, such as tests of normality and adjustment for multiple comparisons
☐	☒	A full description of the statistical parameters including central tendency (e.g. means) or other basic estimates (e.g. regression coefficient) AND variation (e.g. standard deviation) or associated estimates of uncertainty (e.g. confidence intervals)
☐	☒	For null hypothesis testing, the test statistic (e.g. F, t, r) with confidence intervals, effect sizes, degrees of freedom and P value noted<br>*Give P values as exact values whenever suitable.*
☒	☐	For Bayesian analysis, information on the choice of priors and Markov chain Monte Carlo settings
☒	☐	For hierarchical and complex designs, identification of the appropriate level for tests and full reporting of outcomes
☒	☐	Estimates of effect sizes (e.g. Cohen's d, Pearson's r), indicating how they were calculated

Our web collection on statistics for biologists contains articles on many of the points above.

Software and code

Policy information about availability of computer code

Data collection	Data collection was performed employing EthoVision XT (v.11.5) by Noldus, Olympus slide scanner VS120, Nikon Eclipse Ti2, LSM800 ZEN Blue imaging software by Zeiss (v.2.3), Proteomics was done using a Orbitrap Fusion Lumos mass spectometer controlled by Xcalibur 4.1 software (v.4.1) and quantified using Proteome discoverer (v.2.1) by Thermo Scientific, FACS sorting was performed on a BD FACS Aria III, Western blots were imaged on a GE Healthcare Amersham imaging machine, RT-qPCRs done on the Roche Lightcycler 480, electrophysiological data was acquired using a MultiClamp 700B amplifier and Digidata 1550A and Clampfit 10 (v10.7) software (Molecular Devices), image analysis was done in Imaris - Microscopy Image Analysis Software by Bitplane (v.9.3.1), in Fiji (v.1.50e and 1.52n) including the Trackmate, Manual Tracking and Cell Counter plugins, random migration assays and time-lapse live-imaging were analysed using the Ibidi chemotaxis and Migration Tool (v.2.0), STED images were acquired on an Abberior Instruments Expert Line STED microscope.
Data analysis	Statistical analyses were performed in Excel 2013, Origin Software (Origin Inc.), GraphPad Prism 6 and 8, R statistical programming language using in house scripts and the limma package, GO-term analysis was done using DAVID Bioinformatics Resources 6.8 online tool, cell migration was analysed using the ibidi Chemotaxis and Migration tool V2.0, SiR-actin STED images were analyzed using custom-written Phyton routines using the scikit-image library (version 0.16.2) and the scipy library (version 1.3.0).

For manuscripts utilizing custom algorithms or software that are central to the research but not yet described in published literature, software must be made available to editors/reviewers. We strongly encourage code deposition in a community repository (e.g. GitHub). See the Nature Research guidelines for submitting code & software for further information.

Data

Policy information about availability of data

All manuscripts must include a data availability statement. This statement should provide the following information, where applicable:

- Accession codes, unique identifiers, or web links for publicly available datasets
- A list of figures that have associated raw data
- A description of any restrictions on data availability

All data is available from the corresponding author upon reasonable request.
Mouse mass spectrometry proteomics data are deposited at the ProteomeXchange Consortium via the PRIDE 46 partner repository with the dataset identifier "PXD017040". Figures with associated raw data are Figure 5b-h and Supplementary Figure 8b-fd, i-j.
Publicly available datasets used include the Allen Cell Types Database [https://portal.brain-map.org/atlases-and-data/rnaseq] and the BrainSpan Atlas [http://www.brainspan.org/static/download.html]. Figures with associated data are Supplementary Figure 3d-g.

Field-specific reporting

Please select the one below that is the best fit for your research. If you are not sure, read the appropriate sections before making your selection.

☒ Life sciences ☐ Behavioural & social sciences ☐ Ecological, evolutionary & environmental sciences

For a reference copy of the document with all sections, see nature.com/documents/nr-reporting-summary-flat.pdf

Life sciences study design

All studies must disclose on these points even when the disclosure is negative.

Sample size	Sample size for each experiment is indicated in the figure legend for each experiment. The sample size for behavioral tests was predetermined using the tool provided here: http://biomath.info/power/ttest.htm. In addition, calculated sample size was compared against sample sizes known from previous experience and in relevant literature. Accordingly, and given the high variability of behavioral data, in some of the tests we decided to use less animals, since the generated numbers were ethically unacceptably high. No statistical methods were used to pre-determine sample sizes for our histological, cellular an proteomics experiments, but our sample sizes are similar to those reported in previous publications (Deliu et al. 2018, Tărlungeanu et al. 2016, Gstrein et al. 2018).
Data exclusions	Exclusion criteria have always been pre-established and respected throughout data acquisition and analysis. Examples of general exclusion criteria: death of an animal during behavioral testing let to the exclusion of its littermate sibling; in the in vitro neurosphere assay, spheres too close to the well's border were excluded due to limited migration radius; as previously described (Guenthner et al. 2013), in 5-20% of tamoxifen injected CreER mice recombination fails, therefore we checked Cul3 protein levels at the end of the experiments and data from animals in which Cul3 protein levels had not sufficiently decreased were excluded. No outlier was removed from any data set with the exception of one trace aquired in a GFP-labeled neuron in the cortex of a Cul3 embryo, which clearly clustered away and was excluded after outlier analysis (GraphPad Prism 8, Q=0.5%) and was therefore excluded from analysis; as well as one sample in the adult proteomics analysis (hippocampus and cortex samples, therefore n= 4) that clustered clearly away from all other samples (clustering by PCA) during the preliminary quality assessment, samples from this mouse and its corresponding littermate sibling were excluded from further analyses.
Replication	To ensure that our experimental findings can be reliably replicated, each experiment was repeated in multiple behavioral cohorts or experimental replicates (each experiment was at least repeated twice). All attempts at replication were successful.
Randomization	For in vivo experiments, mice were chosen based on genotypes. Sex-matched animal pairs of control-mutant siblings from the same litters were compared to decrease variance in age, environment and genetic background. At least 5 litters of animals were used in each behavioral test. Females and male cohorts were tested and analyzed separately initially; data was only pooled after confirming that female and male wild-type animals did not significantly defer, else the data for each sex is presented separately. The sex-depenent differences in the parameters analyzed were not the purpose of the present study, however, in the set of experiments employed here, the discrepancies were minimal. In all experiments, animals were assigned for groups while controlling for variability in circadian rhythms.<br>Independent experiments were performed across different batches of cells. Cul3+/+ and Cul3+/- NPCs were matched according to the same batch and to a similar passage number.
Blinding	Both data acquisition and analysis were performed blind to the genotype/ experimental condition.

Reporting for specific materials, systems and methods

We require information from authors about some types of materials, experimental systems and methods used in many studies. Here, indicate whether each material, system or method listed is relevant to your study. If you are not sure if a list item applies to your research, read the appropriate section before selecting a response.

Materials & experimental systems

n/a	Involved in the study
	x Antibodies
	x Eukaryotic cell lines
x	Palaeontology
	x Animals and other organisms
x	Human research participants
x	Clinical data

Methods

n/a	Involved in the study
x	ChIP-seq
x	Flow cytometry
x	MRI-based neuroimaging

Antibodies

Antibodies used

As described in the Methods section, the following antibodies were used:
- rat monoclonal anti-BrdU (BioRad, MCA2060T, 1:500)
- rabbit polyclonal anti - cl. Caspase3 (Cell Signaling, 9661, 1:300)
- rabbit monoclonal anti-GFAP (Cell Signaling, 12389, 1:300)
- rabbit polyclonal anti-Cux1 (Santa Cruz, sc-13024, lot #C2615, 1:200)
- rat monoclonal anti-Ctip2 (Abcam, ab18465, 1:500)
- mouse monoclonal anti-Parvalbumin (PV) (1:500, Chemicon MAB1572)
- rabbit polyclonal anti-Iba1 (1:500, Wako 019 19741)
- rabbit polyclonal anti-Sox2 (1:200, Millipore, AB5603)
- rabbit monoclonal anti-Tbr2 (1:250, Abcam AB183991)
- rabbit polyclonal anti-Phospho-Histone H3 (1:500, Millipore 06-570)
- rabbit polyclonal anti-Gapdh (1:1000, Merck, ABS16)
- mouse monoclonal anti-beta actin (1:1000, Sigma A1978)
- rabbit polyclonal anti-Pls3 (1:500, Thermo Fisher, PA5-27883)
- rabbit polyclonal anti-Pls3 (1:1000, Proteintech, 12917-1-AP)
- mouse monoclonal anti-Pls1 (1:500, Novus Biologicals, H00005357-M04)
- rabbit monoclonal anti-INA (1: 5000, Abcam, ab40758)
- mouse monoclonal anti-Nischarin B3 (1:200, Santa Cruz, sc-365364)
- rabbit polyclonal anti-Cul3 (1:800, Cell Signaling, #2759)

Validation

Antibodies used in this study were validated by the manufacturer who provided references on their websites using the catalog number provided and/or proven to work in the following papers (references below):
- rat monoclonal anti-BrdU (BioRad, MCA2060T, 1:500) was previously used in many publications to stain for proliferation of cells in the mouse developing brain after a BrdU pulse (refs. 1-4).
- rabbit polyclonal anti - cl. Caspase3 (Cell Signaling, 9661, 1:300) raised against amino-terminal residues adjacent to (Asp175) in human caspase-3. It has been used to study cell death in mouse brain (refs. 1,5).
- rabbit monoclonal anti-GFAP (Cell Signaling, 12389, lot# 4, 1:300) from rabbits immunized with a synthetic peptide corresponding to residues surrounding Asp395 of human GFAP protein. The antibody was shown in western blot (WB) analysis by the manufacturer to specifically bind GFAP in mouse and rat brain lysates and used in immunoflourescent stainings (IF) to detect astrocytes in spinal cord organotypic cultures. With our immunostainings, we were able to detect the characteristic staining pattern of GFAP. The signal marked processes near the ventricle, in the hippocampus, throughout the cortex, and near the pial membrane (refs. 1,6).
- rabbit polyclonal anti-Cux1 (Santa Cruz, sc-13024, lot #C2615, 1:500) against aa 1111-1332 (c-terminus) of mouse CDP (Cux1). This antibody was previously used to stain the upper cortical layers (II-III) in a study of cortical development. In our experiments we were also able to find this expected laminar staining pattern of the upper cortical layers (refs. 1,7).
- rat monoclonal anti-Ctip2 (Abcam, ab18465, 1:500) antibody raised against a fusion protein corresponding to Human Ctip2, aa 1-150. This antibody was previously used to detect Ctip2- positive neurons during mouse brain development but also in the adult brain (refs. 1,8,9).
- mouse monoclonal anti-Parvalbumin (PV) (1:500, Chemicon MAB1572) antibody raised in mouse IgG1 against PV purified from frog muscle (clone PARV-19). It was previously used in immunoflourescent stainings to identify PV+ fast-spiking interneurons in the visual cortex (ref. 10).
- rabbit polyclonal anti-Iba1 (1:500, Wako 019 19741) antibody against ionized calcium-binding adapter molecule 1 expressed in microglial cells. It has previously been used to identify microglia in the mouse brain (ref. 11).
- rabbit polyclonal anti-Sox2 (1:200, Millipore, AB5603) antibody raised against a KLH-conjugated linear peptide corresponding to a C-terminal region sequence of human Sox2. This antibody has previously been used to identify radial glia cells in the developing cortex of mouse embryos (ref. 12).
- rabbit monoclonal anti-Tbr2 (1:250, Abcam AB183991) antibody produced recombinantly using a recombinant fragment within Mouse TBR2/ Eomes aa 250-500. It has been validated in several publications to identify intermediate progenitors in the mouse cortex (ref. 18)
- rabbit polyclonal anti-Phospho-Histone H3 (1:500, Millipore 06-570) for detection of Histone H3 phosphorylated at serine 10. It has previously been validated to identify mitotic cells in M-Phase (ref. 19).
- rabbit polyclonal anti-Gapdh (1:1000, Merck, ABS16) antibody raised against a GST-tagged recombinant protein corresponding to the C-terminus of human GAPDH. It has previously been used as a loading control for western blot with mouse tissue lysates (ref. 13).

- rabbit polyclonal anti-Pls3 (1:500, Thermo Fisher, PA5-27883) antibody raised against a recombinant fragment corresponding to a region within amino acids 66 and 371 of Human T-Plastin. The antibody has been shown by the manufacturer to specifically bind mouse Pls3 in whole cell extracts from mouse embryonic fibroblasts in western blot analysis.
- rabbit polyclonal anti-Pls3 (1:1000, Proteintech, 12917-1-AP) against a PLS3 fusion protein Ag3574. It was previously shown by the manufacturer to detect human Pls3 in lysates from a A431 cell line in western blot.
- mouse monoclonal anti-Pls1 (1:500, Novus Biologicals, H00005357-M04) against a PLS1 (aa 1 - 102) partial recombinant protein with GST tag. It has been previously used to detect mouse Pls1 by western blot (ref. 14).
- rabbit monoclonal anti-INA (1: 5000, Abcam, ab40758) against a synthetic peptide within Human alpha-Internexin aa 400-500. It has previously been used to detect alpha-internexin in mouse brain lysates (ref. 15).
- mouse monoclonal anti-Nischarin B3 (1:200, Santa Cruz, sc-365364) against the amino acids 539-567 of human Nischarin. It has previously been used to detect Nischarin by western blot in mouse brain tissue (ref. 16).
- rabbit polyclonal anti-Cul3 (1:800, Cell Signaling, #2759) antibody raised against residues surrounding Leu750 of human Cullin-3. It has been previously validated to specifically bind Cul3 in mouse tissue (ref. 17).

1. Deliu, E. et al. Haploinsufficiency of the intellectual disability gene SETD5 disturbs developmental gene expression and cognition. Nat. Neuroscience 21, pages1717–1727(2018).
2. Takeo, Y., Kurabayashi, N., Nguyen, M.D. & Sanada, K. The G protein-coupled receptor GPR157 regulates neuronal differentiation of radial glial progenitors through the Gq-IP3 pathway. Sci Rep 6, 25180 (2016).
3. Kaslin, J., Kroehne, V., Ganz, J., Hans, S. & Brand, M. Distinct roles of neuroepithelial-like and radial glia-like progenitor cells in cerebellar regeneration. Development 144, 1462-1471 (2017).
4. Voronova, A., et al. Migrating Interneurons Secrete Fractalkine to Promote Oligodendrocyte Formation in the Developing Mammalian Brain. Neuron 94, 500-516 e509 (2017).
5. Stefani, J., et al. Disruption of the Microglial ADP Receptor P2Y13 Enhances Adult Hippocampal Neurogenesis. Front Cell Neurosci 12, 134 (2018).
6. Haan, N., Zhu, B., Wang, J., Wei, X. & Song, B. Crosstalk between macrophages and astrocytes affects proliferation, reactive phenotype and inflammatory response, suggesting a role during reactive gliosis following spinal cord injury. J Neuroinflammation 12, 109 (2015).
7. Jeong, S.J., et al. GPR56 functions together with alpha3beta1 integrin in regulating cerebral cortical development. PLoS One 8, e68781 (2013).
8. Zhou, W., et al. BLOS2 negatively regulates Notch signaling during neural and hematopoietic stem and progenitor cell development. Elife 5 (2016).
9. Nikouei, K., Munoz-Manchado, A.B. & Hjerling-Leffler, J. BCL11B/CTIP2 is highly expressed in GABAergic interneurons of the mouse somatosensory cortex. J Chem Neuroanat 71, 1-5 (2016).
10. Cooke, S.F. et al. Visual recognition memory, manifested as long-term habituation, requires synaptic plasticity in V1. Nature Neuroscience 18, 262-71 (2015).
11. Qin, Y. et al. A Milieu Molecule for TGF-β required for Microglia Function in the Nervous System. Cell 174, Issue 1, 18-20 (2018).
12. Caballero, I.M. et al., Cell-autonomous repression of Shh by transcription factor Pax6 regulates diencephalic patterning by controlling the central diencephalic organizer. Cell Rep. 8(5):1405-18 (2014).
13. Chen, F.Q. et al., Traumatic noise activates Rho-family GTPases through transient cellular energy depletion. JNeurosci. 32 (36) 12421-12430 (2012).
14. Krey, J.F. et al., Plastin 1 widens stereocilia by transforming actin filament packing from hexagonal to liquid. J Cell Biol. 215 (4):467-482 (2016).
15. Iqbal, J. et al., Selenium positively affects the proteome of 3 × Tg-AD mice cortex by altering the expression of various key proteins: unveiling the mechanistic role of selenium in AD prevention. J. Neurosci. Res. 96:1798-1815 (2018).
16. Gstrein, T., et al., Mutations in Vps15 perturb neuronal migration in mice and are associated with neurodevelopmental disease in humans. Nat. Neuroscience 21(2):207-217 (2018).
17. Singer, J.D., et al., Cullin-3 targets cyclin E for ubiquitination and controls S phase in mammalian cells. Genes Dev. 13 (18):2375-87 (1999).
18. Kawaue T, Shitamukai A, Nagasaka A, Tsunekawa Y, Shinoda T, Saito K, Terada R, Bilgic M, Miyata T, Matsuzaki F, Kawaguchi A. Lzts1 controls both neuronal delamination and outer radial glial-like cell generation during mammalian cerebral development. Nat Commun. 2019 Jun 25;10(1):2780.
19. Otsuki L, Brand AH. Cell cycle heterogeneity directs the timing of neural stem cell activation from quiescence. Science. 2018 Apr 6;360(6384):99-102

Eukaryotic cell lines

Policy information about cell lines

Cell line source(s)	The Cul3+/+ and Cul3+/- neuro-progenitor cell lines (NPCs) used in this study were generated in house from the E13.5 mutant and wild-type embryos (see methods). The B16-F1 mouse melanoma cells were obtained from ATCC® (CRL-6323TM). Lenti-X™ 293T cell line was obtained from Takara (cat. nr. 632180).
Authentication	Mouse NPCs were not authenticated, B16-F1 cells were authenticated by ATCC, Lenti-X™ 293T were authenticated by Takara. Authentication procedures were not explicitly described by the manufacturer.
Mycoplasma contamination	Mouse NPCs were not tested for mycoplasm contamination; B16-F1 and Lenti-X™ 293T cells were tested negative for mycoplasm contamination.

Commonly misidentified lines (See ICLAC register)	No misidentified cell lines were used in this study.

Animals and other organisms

Policy information about studies involving animals; ARRIVE guidelines recommended for reporting animal research

Laboratory animals	C57BL/6J, Cul3+/-, Cul3flox mice crossed to the Emx1-Cre or Cag-CreER lines, male and female mice with age between E14.5 and P150 were used in the present study.<br><br>Mice were housed in commercially available individually ventilated cages (IVCs) made of Polysulfon under precisely defined standard laboratory conditions (room temperature 22 ± 1 °C; relative humidity 55 ± 10 %) in groups of 3-4 animals per cage and kept on a 12 hour light/dark cycle (lights on at 7:00 am), with food and water available ad libitum.
Wild animals	The study did not involve wild animals.
Field-collected samples	The study did not involve samples collected from the field.
Ethics oversight	All animal protocols were approved by the Institutional Animal Care and Use Committee at IST Austria and the Bundesministerium für Bildung, Wissenschaft und Forschung, Austria.

Note that full information on the approval of the study protocol must also be provided in the manuscript.
